# Development of artificial blood vessels through tissue engineering

**DOI:** 10.1186/1753-6561-8-S4-P45

**Published:** 2014-10-01

**Authors:** Guilherme Colla, Luismar Marques Porto

**Affiliations:** 1 Chemical Engineering Graduate Program - UFSC, Florianópolis-SC, Brazil

## Background

Cardiovascular diseases are one of the main causes of mortality in many parts of the world, with atherosclerosis figuring as the principal cause of coronary occlusion, stroke and aortic aneurysm [1]. Saphenous vein is the most commonly used vascular prosthesis for small-caliber (< 6 mm) vascular grafts, however, 10%-40% of patients do not have a saphenous vein suitable for prosthetic replacement due to size mismatch or venous disease [2]. Tissue engineering approaches are being used to develop palliative methods for these pathologies such as the construction of artificial blood vessels. The purpose of this study was to biosynthesize artificial vessels using *Gluconacetobacter hasenii*'s bacterial cellulose (BC) as scaffolding [3]. Functional and structural characteristics of the vessels were evaluated, as well as the coating of the cellulolytic tubular scaffolding with human aortic smooth muscle cells (HASMC). The vessels obtained exhibited appropriate mechanical properties and their morphology showed connected nonporous and porous phases as a basis to mimic, respectively, the intimal and medial layers of a blood vessel. *In vitro *cultures of HASMCs in the presence of tubular scaffolds demonstrated their ability to support colonization by human aortic smooth muscle cells.

## Methods

BC vessels were developed using *G. hansenii *(ATCC 2376) grown in a mannitol, yeast extract and peptone medium, in an Erlenmeyer flask, containing a silicone tube (4 mm inside diameter) connected to a circulation air pump. Bacterial cells were cultured for 12 days under static conditions and allowed BC biosynthesis around the template tube. The artificial vessels were then washed with NaOH (0.1 mol L^-1^) sterilized by autoclaving. To assess the effect of the artificial device on human cell viability, primary cultures of HASMC were grown over the BC vessels, in a 231 medium at 37ºC with 5% of CO_2_. Vessels' morphology was evaluated by scanning electron microscopy (SEM) and the mechanical properties of the vessels assessed by tensile tests. For the viability assays, cells were seeded at a concentration of 15 × 10^5 ^cell/vessel, grown in the nonporous side of the membrane and analyzed by MTS colorimetric assay [3-(4,5-dimethylthiazol-2-yl)-5-(3-carboxymethoxyphenyl)-2-(4-sulfophenyl)-2H-tetrazolium], after 24, 48, 72h and 7, 14 days of culture. The absorbance was quantified at 490 nm in a microplate reader.

## Results and conclusions

SEM analysis showed that BC vessels are composed of fibers with a nonporous surface formed at the liquid/solid/air interface (inside the vessel), followed by a porous layer (outside the vessel). Tensile tests showed similar burst strength of the cellulose vessel when compared to a native blood vessel (224 ± 41 kPa). Human cells remained viable over the BC scaffolding for up to 14 days, the total duration of the experiment indicating that BC vessels represent a suitable support for cell colonization. Results presented here highlight the potential of BC as a biomaterial for vascular tissue engineering and its applications as a model for artificial blood vessels.

## References

[B1] Barron V Lyons E Stenson-Cox C Mchugh PE Pandit A Bioreactors for Cardiovascular Cell and Tissue Growth: A Review Ann Biomed Eng 2003 31 1017 1030 14582605 10.1114/1.1603260

[B2] Conklin BS Richter ER Kreutiziger KL Zhong DS Chen C Development and evaluation of a novel decelularized vascular xenografts Med Eng Phys 2002 24 173 183 10.1016/S1350-4533(02)00010-3 12062176

[B3] Oliveira VA Rambo CR Porto LM Produção e Degradação in vitro de Estruturas Tubulares de Celulose Bacteriana Polímeros 2013 23 559 564

